# Information flow between global financial market stress and African equity markets: An EEMD-based transfer entropy analysis

**DOI:** 10.1016/j.heliyon.2023.e13899

**Published:** 2023-02-22

**Authors:** Mohammed Armah, Ahmed Bossman, Godfred Amewu

**Affiliations:** a Department of Accounting and Finance, School of Business, Ghana Institute of Management and Public Administration (GIMPA), Achimota, Ghana; b Department of Finance, School of Business, CC-191-7613, University of Cape Coast, Cape Coast, Ghana; c Department of Finance, School of Business, University of Ghana, Legon, Ghana

**Keywords:** African stock markets, Global financial market stress, Information flow, Ensemble empirical mode decomposition, Effective transfer entropy, Emerging markets, EEMD, COVID-19 pandemic

## Abstract

The flow of information between markets is important to guide investors and policymakers in the effective allocation of assets and proactive market regulation, respectively. This study examines the impact of information flow from global financial market stress on the African stock markets using the daily US financial stress index (USFSI) and other advanced economies' financial stress index (OAEFSI) to proxy the global financial stress index. To understand the information flow dynamics across various investment horizons, the ensemble empirical mode decomposition (EEMD)-based transfer entropy is employed. Our findings reveal that African equity markets are highly risky for information flow from global financial market stress. However, we identify diversification prospects based on market conditions for Ghana and Egypt in the short term and Tanzania, Cote D'Ivoire, and Egypt in the medium term. Empirical results also show that the information flow from global financial stress to African stock markets depends on time scales, economic relations, and the state of global financial markets. The findings are important for investors, portfolio managers, practitioners, and policymakers.

## 1 Introduction

The negative effect of the subprime crisis that began in the United States between the 2007–2009 period and the current COVID-19 pandemic reverberated on the impact of globalisation on the financial market. The turmoil reminds us that liquidity, shock, insolvency, and losses can easily proliferate and affect international financial markets [1]. The turmoil unleashes a full-blown systemic crisis with increases in global risk aversion and stock markets across countries and regions tumbled as many countries saw their domestic investment decline, causing financial disturbances [[2], [3], [4]]. This reemphasizes the adage that when the United States sneezes, the whole world gets cold. Therefore, it is not surprising that this turmoil has reignited the debate for which the financial stress index has gained prominence in recent times, leading to the proliferation of empirical works. Amewu et al. [2] asserted that policy uncertainties and information associated with the news of policy development could lead to market stress, which affects equity markets. As noted by Davig and Hakkio [3], an increase in financial market stress makes investors more risk-averse, leading to a reduction in investment in financial assets and asset prices.

It is worth noting that the heterogeneity of international financial markets in crisis periods is mainly attributable to the response of investors to intensified information flows [7,[8], [30], [32], [69]]. This suggests why in high-risk and systemic crisis periods, determining how various assets and asset classes respond to risk pressures - on various trading scales or horizons – is important for effective portfolio and risk management [[5], [9], [10], [11], [12], [13], [14]]. African stock markets have been considered the main contender for the inclusion of international portfolio diversification [6]. The development of African equity markets as a destination for foreign investment is gradually integrated into the world, making African stock markets susceptible to global crises [15,16]. However, from the proliferated works on the impact of financial stress on financial markets (see Refs. [[17], [18], [19], [20], [21], [22]]), scanty evidence for African markets. Note that the ability of African stock markets to respond to global pressure depends on accompanying information flows, as the efficient market hypothesis (EMH) and the macroeconomic expectation hypothesis (MEH) espouse [14]. We fill this knowledge gap by documenting how African stock markets respond to the information flow from global financial stress in a multiscale transfer entropy paradigm.

Theoretically, the EMH stipulates that stock prices reflect the information available at the time of pricing [15], suggesting that future economic activity is a prerequisite for the determination of current prices of financial assets [14]. In support of the EMH, the MEH of DeStefano [16] posits that the stock market is forward-looking and therefore economic information reflects asset returns. In this regard, stock prices in Africa can react differently to the information flow from financial stress, and this can cause a change in their usability for diversification on different economic or trading scales.

This discussion highlights the need to decompose original data (signal) into various scale components using mode decompositions that are representative of trading periods. Therefore, to quantify the information flow between global financial stress and African stock markets, we resort to the ensemble empirical mode decomposition (EEMD) approach. The EEMD does well to delineate time series into intra-modal functions (IMFs) to yield true signals devoid of fat tails [26,27]. In so doing, we can determine the true relationship between financial stress and African stock markets on various scales. The present study contributes to the literature as follows.

First, the use of EEMD-based transfer entropy offers the opportunity to understand the extent of information flow between variables on different time scales. Second, the use of EEMD delineates the influence of fat tails on the quantification of information flow and deals with non-linearity and asymmetries that evolve during major events and structural changes in financial markets. Third, we document empirical evidence from a less-represented section of global financial markets. Specifically, our analysis is focused on African stock markets that present likely diversification potentials during crisis periods [28], but have been largely ignored in empirical analysis. Fourth, evidence from this study will inform policymakers about the level of efficiency across economic cycles that affect marketability. Through this, trading horizon-based policies may be facilitated to mitigate the impact of information flow on stock markets during global financial market stress periods.

From the findings, the study reveals that global financial market stress poses a significant risk to African stock markets. The results also show that the flow of information from global financial market stress to African stock markets depends on time scales, economic relations, and the state of global financial markets. Therefore, risk and policy management must be dynamic to accommodate the heterogeneous impacts of financial stress on asset prices and returns.

The rest of the paper is organised as follows. Section 2 presents an empirical background. The methodological steps are outlined in Section 3. In Section 4, data metrics and the statistical properties of the data are described. Section 5 presents the empirical findings and Section 6 concludes.

## 2 Literature review

Within the body of literature, Bianconi et al. [20] examined the behaviour of stocks and bonds from BRIC (Brazil, Russia, India, and China) nations and US financial stress using the VAR and GARCH models. The authors showed that the correlation between stocks, bonds, and US financial stress increased after the collapse of Lehman Brothers. Nyakurukwa [21] examined the information flow between the Zimbabwe Stock Exchange and the Johannesburg Stock Exchange using transfer entropy in a tranquil period. The finding of the result shows that the effective transfer entropy (ETE) values were relatively low, indicating weak links between the paired series. With a slightly different focus, Osei and Adam [22] quantified the information flow between the Ghana stock market index and its constituents. The study found a bidirectional and unidirectional flow of information between the Ghana Stock Exchange index and its component stocks. In the work of Eom et al. [23], the authors concluded that the information flow evidences a property of time dependence which is influenced by the difference in the degree of efficiency in the stock market. Other existing literature within the body of information flow concentrates on economic policy uncertainty (EPU) and stock returns (see [14,24,25]). The findings evidence the relationship between the EPU and the stock returns which is consistent with the EMH and the market expectation hypothesis [35,36].

Due to the complexity of the amount of information that flows from global financial stress (GFS) to the international market, coupled with its adaptive behaviour and heterogeneity of its participants, empirical findings within this body of literature, including those documented by Refs. [37,38] are generally mixed. Furthermore, these studies predominately present analysis based on VAR, GARCH, and entropy models using signal data, which overlooks nonlinear dynamics and offers no evidence at different time scales. The varying results and lack of consensus on the findings in the existing literature could be attributed to methodological inadequacy. A method that quantifies the mutual information shared between financial markets [10,12] is necessary to fill this gap. Therefore, in the present study, we quantify the mutual information between the financial stress index (FSI) and African stock markets on different trading scales.

Theoretically, market participants react to information under different conditions, leading to very noisy market data [[33], [34], [39], [40], [41]]. Lo [36] asserts that market changes due to events and structural breakdowns, which cause the efficiency of the market to oscillate over time. Decomposition of the data reduces weak signals (noise) and maintains exact signals [37]. To overcome the problem of noisy data, which is consistent with the heterogeneous market hypothesis [38], and resolve the methodology drawback in the literature, this study employs the ensemble empirical mode decomposition (EEMD) approach to delineate signal data into IMFs.

The EEMD replaces the empirical mode decomposition (EMD) approach, which suffers from the problem of frequency appearance of mode fixing [39]. The EEMD is completely based on a local time scale without a priori base, and the extracted oscillation accurately reflects the time series [40]. Consequently, in EEMD, we present the transfer entropy proposed by Schreiber [41] to quantify the information flow from global financial market stress to African stock markets. Transfer entropy has been proven to be an effective scalar measure to capture directional and dynamical features between different components of time series [42]. Transfer entropy is very flexible when dealing with symmetric and non-linear data [43]. A recent study by Benthall [28] asserts that information flows are casual flows located in the context of other casual links such that when two random variables are linked, one variable can learn about the state of other variables from observation. Assessing this mutual flow of information provides an indication of financial contagion [35]. Despite the satisfactory attention received in the financial stress-economic activity nexus, quantification of the information flow from GFS to African stock markets using a multiscale transfer entropy remains unexplored, to the best of our knowledge.

Given the relevance of the transfer entropy and decomposition approach in economics and finance [4,25,31,35,42,[44], [45], [46], [47], [48], [49], [50], [51]], it is necessary to employ this novel technique to quantify the flow of information from global financial stress to African stock markets. Although EEMD-based transfer entropy may not reveal the time-varying dynamics of the financial stress-stock markets nexus, its application is essential to delineate signal data into various modes that are representative of investment horizons (short-, medium-, and long-term), which are particularly relevant for market participants and policymakers amid systemic crisis periods. Besides, to overcome the limitation of not showing how the dynamics of information flow may differ across time, we perform subsample analyses to compare how the financial stress-stock markets nexus manifests across different timeframes and investment horizons.

## 3 Methodology

### 3.1 The EEMD approach

We employ Ensemble Empirical Mode Decomposition (EEMD) to decompose the original series into intrinsic mode functions (IMFs). The outputs generated from the EEMD are used as input data for effective transfer entropy (ETE) estimation. The EEMD technique is an improvement of the empirical mode decomposition (EMD) technique, which defines the true IMF components as the mean of a series of tests in which each consists of a signal plus a white noise of finite amplitude [39]. IMFs are found on each scale from satisfactory to coarse using an iterative procedure called the sifting algorithm [52]. The IMFs represent different time series that are relevant in this study given the nonlinearity and non-stationarity within the time series [58]. The decomposition of EMD decomposition is implemented through a sifting process that uses only local extrema [39].

The first condition is to identify the local maxima and connect all these maxima and minima into a cubic spline (lower and upper envelopes), secondly, obtain the component by taking the difference between the series and the local mean of the two envelopes. Repeat the processes until the envelope is symmetric concerning zero mean under certain criteria. At the end of the decomposition process, the original time series of EMD is as follows.

(1) $$s\left(t\right)=\sum_{j=1}^{n}{imf}_{j}\left(t\right)+r_{n}\left(t\right)$$

where *n* = the number of IMFs that are nearly orthogonal to each other and all have zero means and $r_{n}$ is the final residual value with a low-frequency trend of the signal s(t) and is a non-oscillating drift of the data [46,57]. Inferring from Equation (1), the standard deviation is calculated from two consecutive shifting results and is used as a condition to end the shifting process by limiting the standard deviation size as per [53] as follows:

(2) $$SD\left(k\right)=\frac{\sum_{t=0}^{T}{\left[d_{k-1}\left(t\right)-d_{k}\left(t\right)\right]}^{2}}{\sum_{t=0}^{T}d_{k-1}^{2}\left(t\right)}<\varepsilon$$

where *k* is the index of the *kth* difference between the s(t)ande(t), and ε is a pre-determined stopping value.

With the above properties of EMD from equation (2), the proposed EEMD is presented as follows, following Wu and Huang [39]:

Step I Add a white-noise series to the targeted data.

Step II The data were decomposed with added white noise into IMFs and finally Steps I & II with different white noise were repeated to obtain an ensemble of IMFs for the decomposition of the final results.

The effect of decomposition using EEMD is that the added white noise series cancel each other out in the final mean of the ensemble IMFs, the mean IMFs stay within the natural dyadic filter, and thus reduce the chance of mode mixing, which preserves the dyadic property [39]. The effect of adding white noise is to provide a uniform reference frame in the time-frequency space [39].

### 3.2 Rényi transfer entropy

The concept of entropy is rooted in information theory [54] and is based on the concept of Shannon entropy as a measure of information uncertainty [55]. We consider probability distributions where different outcomes occur with a different probability $p_{j}$. Following [56], the average information per symbol is defined as follows:

(3) $$H=\sum_{j=1}^{n}p_{j}\;\log_{2}\left(\frac{1}{p_{j}}\right)bits\text{,}$$

where n is the number of distinct symbols associated with the probabilities $p_{j}$. The base of the logarithm from Equation (3) determines the unit used to measure the information. The concept of entropy by Shannon [57] asserts that for a discrete random variable *j* with the probability distribution $p_{j}$, the average number of bits required to optimally encode the draw independent is as follows;

(4) $$H_{j}=-\sum_{j=1}^{n}p_{j}\;\log_{2}p\left(j\right)\text{.}$$

The concept of Shannon entropy is connected to Kullback. & Leibler's [58] distance, under the assumption that underlines Markov processes, where *i* and *j* denote two discrete random variables with marginal probability $p_{i}$ and $p_{j}$ joint probability p(i,j) with dynamic structures in line with the stationary Markov process of *k* (process *1*) and *i*(process *j*). The Markov process infers that the probability of observing *i* at time t+1 in the state *is* conditional on the previous observation is ($i_{t+1}|i_{t}\ldots \ldots i_{t-k+1})$ = ($i_{t+1}|i_{t}\ldots \ldots i_{t-k-1})$. To encode the observation in *t+1* from Equation (4), the average number of bits that require the previous value k is given by

(5) $$H_{j}(k)=-\sum_{i}^{n}p(i_{t+1,i_{t}^{\left(k\right)}})logp\left((i_{t+1,|\;i_{t}^{\left(k\right)}}\right)\text{.}$$

where $i_{t}^{\left(k\right)}$ = ($i_{t+1}|i_{t}\ldots \ldots i_{t-k+1})$ analogously for processes j. Inferring from Equation (5), the information flow from process *i* to *j* is measured by quantifying the deviation from the generalised Markov property $p\left(i_{t+1,i_{t}^{\left(k\right)}}\right)$ = $\left(i_{t+1,i_{t}^{\left(k\right)}}\right)$, $j_{j}^{i}$, relying on the concept of Kullback. & Leibler distance, the Shannon transfer is presented as follows:

(6) $$T_{j\to i}\left(k,l\right)=\sum p\left(i_{t+1,i_{t}^{\left(k\right)}}j_{j}^{i}\right)\log\frac{p\left(i_{t+1,i_{t}^{\left(k\right)}}j_{j}^{i}\right)}{p{(i}_{t+1,i_{t}^{\left(k\right)}})}$$

where $T_{j\to i}$ measure information flow from *j* to *i*. $T_{i\to j}$ measure information flow from *i* to j. The dominant direction of the information flow can be determined by calculating the difference $T_{j\to i}$ and $T_{i\to j}$. The transfer entropy can also be based on the Rényi entropy [59], which depends on the weighted parameter q and can be calculated as follows [60];

(7) $$H_{j}^{q}=\frac{1}{1-q}\log\sum_{j}p^{q}\left(j\right)$$

With q > 0, for q > 1 Rényi entropy converts to Shannon entropy as follows:

(8) $$H_{J}=\log\sum_{j}p\left(j\right)logp\left(j\right)$$

For 0< q < 1, the event with low probability receives more weights, *q > 1,* weight favours the outcome j with higher initial probability. Inferring from Equations (7), (8), allows the stress of different areas of distribution to be applied, depending on the parameter *q* [54].

Using the escort distribution Beck [61] $\emptyset_{q}\left(j\right)=(p^{q}(j)/\sum_{j}p^{q}\left(j\right)$ with q > 0 to normalise the weighted distribution. The Rényi transfer entropy is derived as follows [60]:

(9) $${RT}_{j\to i}\left(k\;l\right)=\frac{1}{1-q}\log\frac{\sum_{i}\emptyset_{q}(i_{t}^{\left(k\right)}p^{q}(i_{t+1,|\;i_{t}^{\left(k\right)}})}{\sum_{i,j}\emptyset_{q}\left(i_{t}^{\left(k\right)},j_{t}^{\left(l\right)}\right)p^{q}\left(i_{t+1},|i_{t}^{\left(k\right)},j_{t}^{\left(l\right)}\right)}$$

Inferring from Equation (9), it is important to note that the calculation of the Rényi transfer entropy can have negative results; therefore, knowing the history of the real values of j can have even greater uncertainty than would otherwise be indicated by only knowing the history of *i* [60]. Transfer entropy (ETE) estimates are usually biased when the sample size is small [68]. To address this bias, we calculate the effective transfer entropy as follows [62];

(10) $${ET}_{j\to i}\left(k,l\right)=T_{j\to i}\left(k,l\right)-T_{shuffled\to }\left(k,l\right)$$

From equation (10), $T_{shuffled\to i}\left(k,l\right)$ illustrates the transfer entropy using a shuffled version of the time series *j*. Shuffling involves randomly drawing values from the observed time series *j* and rearranging them to generate a new time series. Therefore, this destroys the time series dependency of *j,* as well as the statistical dependencies between *j* and *i.* consequences $T_{shuffled\to i}\left(k,l\right)$ converges to zero with increasing sample size, and any nonzero value of $T_{shuffled\to }i\left(k,l\right)$ is due to its small size. To deal with bias-corrected effective transfer entropy estimates, the shuffling is repeated, and the average of the resulting shuffled transfer entropy estimate overall replication indicates a predictor for the small-sample bias, which is subsequently removed from the Shannon or Rényi entropy estimate to obtain a bias-corrected effective transfer estimate.

To order the statistical significance of the entropy estimate as provided in Equation (6), the Markov block bootstrap was used [63]. Unlike shuffling, it conserves the dependence within variables *j* and *i,* but removes the statistical dependencies between them. Under the null hypothesis of no information flow, the bootstrapping provides a distribution of the transfer entropy estimate with the associated probability value given by 1-${\hat{q}}_{T}$. Where ${\hat{q}}_{T}$ specifies the quantile of the simulated distribution, that is, determine by transfer entropy estimates [54].

The transfer entropy estimate technique is based on discrete data. Therefore, a continuous date must be discretised. This is done by dividing the data into a finite number of bins. This technique is called symbolic encoding [54]. The symbolically encoded time series for a quota bins *n* and bounds $q_{1,}q_{2}\ldots ..q_{n-1}$ where $q_{1,}<\;q_{2,}<\;q_{n-1,}$ and consider an observe time series data $y_{t}$ is presented as follows:

(11) $$S_{t}=\left\{ \begin{array}{c} A\;y_{t}\leq q_{0.05}^{r}\\ B\;y_{t}<\;q_{0.05}^{r}\\ C\;y_{t}\geq q_{0.05}^{r} \end{array}\right.<\;y_{t}\leq q_{0.05}^{r}$$

The symbolic encoding replaces each value in the observed returns series $y_{t}$ by the corresponding (A, B, C). The sample was recorded using three bins and the data was divided into quantiles of 5% and 95% [29]. This denoted as $q_{\left(0.05\right)}^{r}$ and $q_{\left(0.95\right)}^{r}$ of the corresponding distribution (lower and upper bounds) of the bins. Inferring from Equation (11), three symbolic encodings are given: the negative extreme (lower tail) is in the first bin (0.05), the positive extreme (upper tail) is in the three-bin (0.95), and the second bin (0.90) is intermediate. Using the chain rule to derive the conditional probability as a fraction of the joint probabilities, the probability in Equations (5), (6) is computed by the relative frequency of all the likely outcomes.

## 4 Data metrics and descriptive statistics

### 4.1 Data metrics

The data considered in this study are the daily series of the Financial Stress Index of the Office of Financial Research (OFR). The OFR FSI has more than 30 indicators with a summary of financial stress, which is decomposed into five indicators, namely, (i) credit measures credit spread, which presents the difference in borrowing cost for firms of different creditworthiness; (ii) equity valuation, which contains stock valuations from several stock market indexes that reflect investor confidence and risk appetite; (iii) funding, which measures how a financial institution can fund its activities; (iv) safe assets, which measure how assets are considered a store of value or have predictable cash flows; and (v) volatility, which measures implied and realised volatility in equity, credit, currency, and commodity markets. We employed OFR FSI because, unlike other FSIs where the entire time series are re-estimated each time, they are updated, and thus OFR FSI respects the arrow of time. This implies that the value on each given day depends only on the information available on that day and, once estimated, its value does not change [64]. We used FSIs from the US and other advanced economies other than the US, including primarily the eurozone and Japan, because financial stress reflects a significant degree of general financial condition in the global financial market [65].

The objective of using stress indices is that the FSI provides detailed information on the financial situation and helps identify latent vulnerabilities underlying weaknesses in the financial system that originate or transmit stress [64]. According to Monin [64], FSI is a monitoring tool that helps predict declines in economic activity. We believe that the FSI offers more insight into changes in investor expectations and captures the dynamics in capital markets.

Data on African stock prices were obtained from DataStream. The sample period covers from January 04, 2011 to March 10, 2022, giving us a total daily observation of 2814 for each series. We further disaggregate the data into sub-samples to establish whether the flow of information from global financial stress to the African stock markets amid uncertainty about global economic policy is driven mainly by the COVID-19 pandemic shock. Therefore, we take the insight from the announcement of a pandemic by the World Health Organisation on March 11, 2020, as a global pandemic, as a basis for splitting our data into the full sample and sub-sample (pre-COVID-19 and COVID-19 periods). Daily stock prices were transformed by taking the logarithmic difference between consecutive prices given as $r_{t}=\left[In\left(P_{t}\right)-In\left(P_{t-1}\right)\right]*100$; where $r_{t}$ is return at the time *t,* $P_{t}$ and $P_{t-1}$ are respectively, current price/index and one-period lagged price/index.

### 4.2 Descriptive statistics

The descriptive statistics presented in Table 1 indicate that the series exhibited leptokurtic behaviour indicating high peakedness with significantly fatter tails. All the series deviate from normality, as depicted by the Jarque-Bera normality statistics. The average daily stock returns for Ghana, Ivory Coast, Morocco, Nigeria, South Africa, and Tanzania were negative in the COVID-19 pandemic sample, while those for Egypt, Ghana, South Africa, Tanzania, and Uganda were negative for the pre-COVID-19 pandemic sample. Similarly, negative returns for USFSI and OAEFSI were recorded both before and during COVID-19. This suggests plausible similar movements between global financial stress and African stock returns. Skewness statistics for the series were all positive, except for Morocco and Nigeria, which exhibited negative skewness before and during the pandemic period for Tanzania. Positive skewness signals that most African stock markets recorded more positive returns during the sample period.

**Table 1:** Descriptive statistics.

Full sample	*μ*	σ	*S*	*K*	*JB*	*P*
Egypt	−4.0E-05	0.0156	1.1741	19.5011	32583.85	0.0000
Ghana	−1.7E-04	0.0140	12.3881	735.4006	62988495	0.0000
Ivory Coast	−5.0E-05	0.0087	0.2210	10.7840	7129.633	0.0000
Kenya	3.2E-04	0.0065	0.6336	9.5531	5225.262	0.0000
Mauritius	3.0E-05	0.0099	3.0693	77.3822	653360	0.0000
Morocco	8.0E-06	0.0067	1.5189	29.7248	84853.87	0.0000
Nigeria	−1.9E-05	0.0114	0.0111	7.8539	2763.502	0.0000
South Africa	−2.0E-04	0.0112	0.6981	10.7415	7257.932	0.0000
Tanzania	−4.4E-04	0.0095	1.8901	235.0605	6318074	0.0000
Uganda	−5.6E-06	0.0117	0.3170	17.0216	23107.2	0.0000
OAEFSI	−7.3E-01	1.2139	1.6483	5.7145	2138.879	0.0000
USFSI	−7.5E-01	0.8814	2.0239	9.6907	7172.372	0.0000
Pre-COVID-19	*μ*	σ	*S*	*K*	*JB*	*P*
Egypt	−0.0002	0.0152	0.3829	10.6646	5710.8	0.0000
Ghana	−0.0002	0.0152	11.8457	649.1438	40238569.0	0.0000
Ivory Coast	0.0000	0.0089	0.1851	10.9676	6123.4	0.0000
Kenya	0.0002	0.0063	0.1491	7.8609	2282.8	0.0000
Mauritius	0.0000	0.0066	0.2985	11.6223	7189.9	0.0000
Morocco	0.0001	0.0058	−0.2037	6.3302	1083.4	0.0000
Nigeria	0.0001	0.0113	−0.1144	6.8419	1425.7	0.0000
South Africa	−0.0002	0.0101	0.2038	4.5070	234.6	0.0000
Tanzania	−0.0005	0.0105	1.7603	196.7981	3616122.0	0.0000
Uganda	−0.7032	1.2121	1.6089	5.5941	1644.3	0.0000
OAEFSI	−0.0001	0.0118	0.1645	17.3485	19826.4	0.0000
USFSI	−0.7188	0.7520	1.3417	5.2347	1173.7	0.0000
COVID-19	*μ*	σ	*S*	*K*	*JB*	*P*
Egypt	0.0008	0.0170	3.7319	44.2498	36975.6	0.0000
Ghana	−0.0001	0.0063	0.6882	15.7413	3455.8	0.0000
Ivory Coast	−0.0005	0.0077	0.4152	8.3743	622.3	0.0000
Kenya	0.0006	0.0075	1.8890	12.4197	2167.4	0.0000
Mauritius	0.0002	0.0189	2.4440	32.6889	19049.5	0.0000
Morocco	−0.0002	0.0097	3.0267	34.0153	21012.0	0.0000
Nigeria	−0.0005	0.0116	0.5484	12.1436	1784.5	0.0000
South Africa	−0.0002	0.0154	1.2429	13.0288	2246.3	0.0000
Tanzania	−0.0001	0.0022	−0.0969	40.8900	30209.3	0.0000
Uganda	0.0004	0.0111	1.1518	14.8807	3081.7	0.0000
OAEFSI	−0.8726	1.2140	1.8692	6.4716	547.7	0.0000
USFSI	−0.8854	1.3131	2.4761	9.2756	1344.7	0.0000

Notes: μ = mean, σ = standard deviation, S = skewness, K = kurtosis, JB = Jarque-Bera, P = probability, USFSI = US financial stress index. OAEFSI = advanced economy financial stress index.

**Fig. 1:**
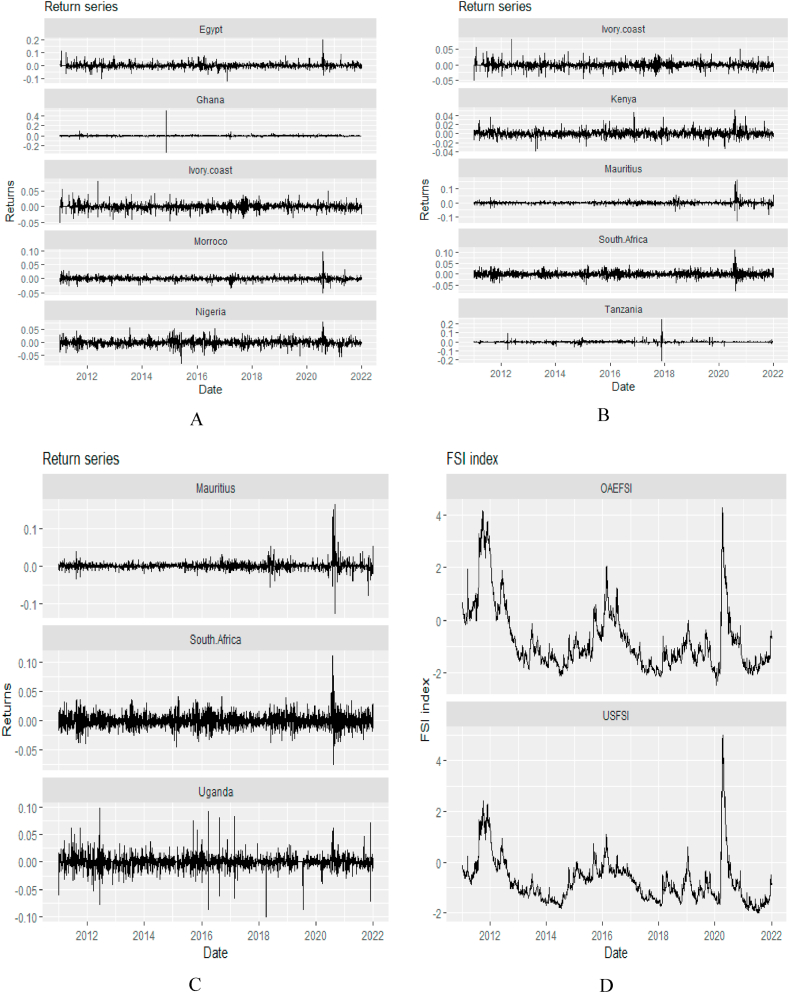
Return series graphs for the US financial stress index (USFSI) and the financial stress index of other advanced economies (OAEFSI). *Notes*: Panels A, B, and C show the plots of return series for African stocks while Panel D shows the time series plots for OAEFSI and USFSI. The sample period falls between January 04, 2011 and March 10, 2022.

Fig. 1 provides plots of return series for African stock markets and the time series plots for the US Financial Stress Index (USFI), and the Financial Stress Index of other advanced economies (OAEFSI). We observe from the plot that the time series plots show stationarity and few spikes with a stable period. We also observe that both USFSI and OAEFSI experienced upward and downward spikes simultaneously in notable crisis periods. The similarity in the spikes between USFSI and OAEFSI supports the position that financial market conditions in the US reflect a significant degree of the overall condition in the global financial market [[71], [72], [73]].

## 5 Empirical results

To shed light on the information flow from global financial stress (GFS) to African equity markets, we present the estimation results for three sample periods to obtain more information on the variability of the information flow over time. The first results deal with the entire sample, followed by samples before COVID-19 and COVID-19. To understand the transfer entropy based on EEMD used in this study, we present the results in the frequency domain by decomposing the time series into IMFs and the residual to obtain an understanding of the scale variability of information flow in African equity markets. This will enable us to analyse the issues of non-stationarity, non-linearity, and asymmetry, which may sometime lead to bedlam [66].

We begin our analysis by decomposing daily returns and indexes to obtain 10 IMFs for the entire sample and pre-COVID-19 and seven IMFs for the COVID-19 pandemic period. The IMFs extracted for this study satisfied all the conditions proposed by Huang et al. [53]. The residual is the non-oscillating drift of the data that presents the structural changes of data generation [67]. Following the existing literature [9,12,13,76], we present the residual as a long-term series, IMFs 1–5 as short-term, and IMFs 6–10 as medium-term. The short term is characterised by investor attitudes and market dynamics [70]; the medium term is characterised by the impact of proceedings [70], and the long term is characterised by fundamental characteristics [77]. In this study, IMFs will help distinguish market dynamics in the context of GFS and are necessary to determine the dynamics of information flow from GFS to African stock markets.

Following the standard practice of the literature, ETEs are shown by black points within the blue bars. The 95% confidence bounds are indicated at the end of the blue bars. If these confidence limits are positive or negative, we reject the null hypothesis of *‘no information flow’.* Information flow is insignificant if there is an intersection at the origin.

### 5.1 Full sample analysis

**Fig. 2:**
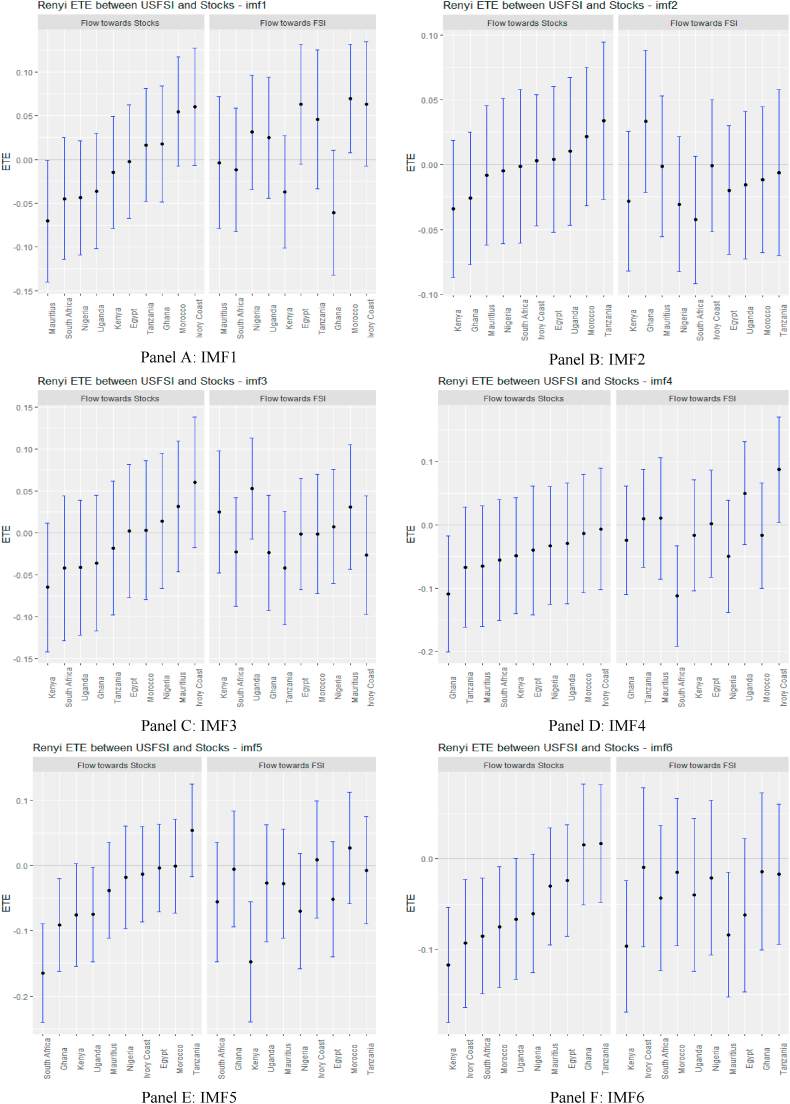

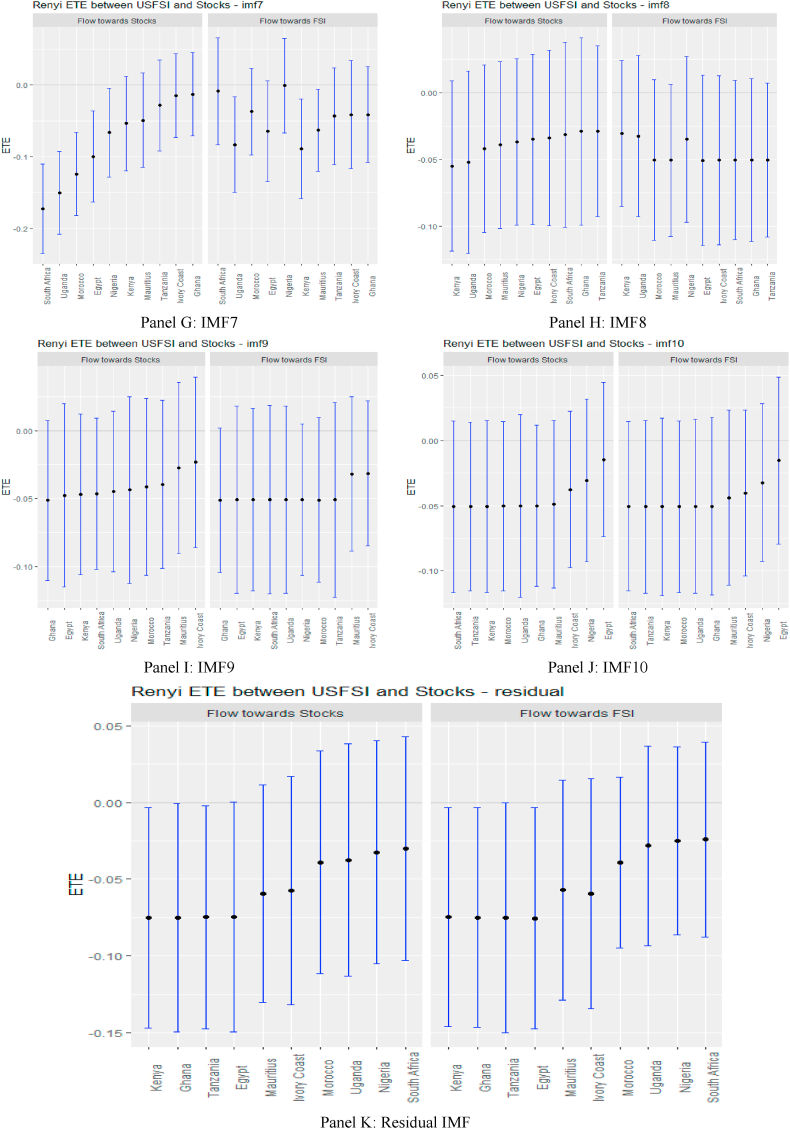
Information flow between the US financial stress and African stock markets (Full sample). *Notes*: Panels A–J show the transfer entropies between the USFSI and African stocks across IMFs1-10, respectively while Panel K is for the residual IMF. The sample period falls between January 04, 2011 and March 10, 2022.

From Fig. 2, supported numerically by the results in Table A1 (see Appendix), we observe that the information flow from USFSI to the African stock markets is insignificant across IMFs 1–5 except Mauritius, Ghana, South Africa and Uganda where the pairs of ETEs are negatively significant, suggesting that stock returns were highly risky within the short term. This implies that the equity market in Mauritius, Ghana, South Africa, and Uganda poses a significantly high-risk investment in the short term. However, since the market participant reacts to the information flow at different times, we observed that from IMF 6–10, global financial market stress (USFSI) transmits negative shocks to Egypt, Ivory Coast, Kenya, Morocco, Nigeria, South Africa, and Uganda. This suggests that the equity market in these economies is highly risky in the medium term. The outcome of information flow from USFSI in the short and medium term is not significantly different from the long term, as several negative receiving transfer entropy increases across the time horizon. This implies a high-risk status for equity markets in Africa in the short, medium and long term such that given the history of market returns from one market, adding more investments from Africa poses a high risk to portfolio diversification. The significant inverse response offered by some equities markets suggests that they could be held as potential diversification in the short term (Mauritius, Ghana, South Africa, and Uganda), in the medium term (Egypt, Ivory Coast, Kenya, Morocco, Nigeria, South Africa, and Uganda), and long term (Egypt, Ghana, Kenya and Tanzania).

**Fig. 3:**
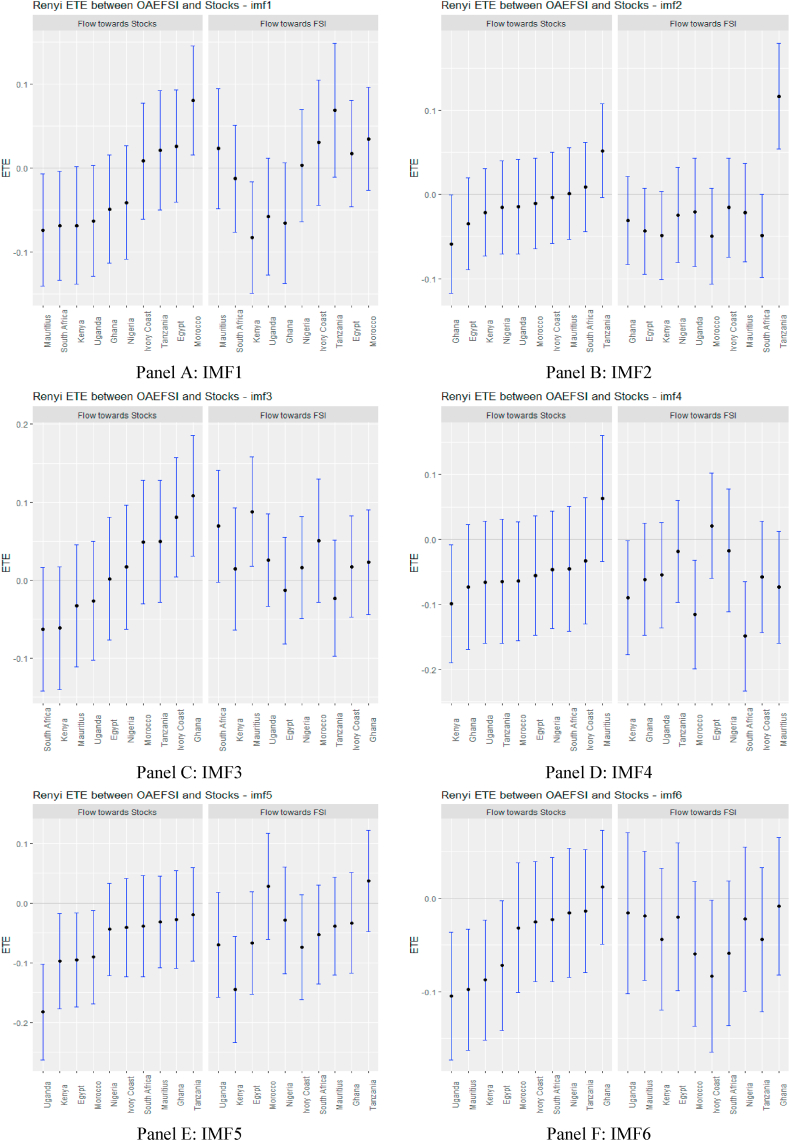

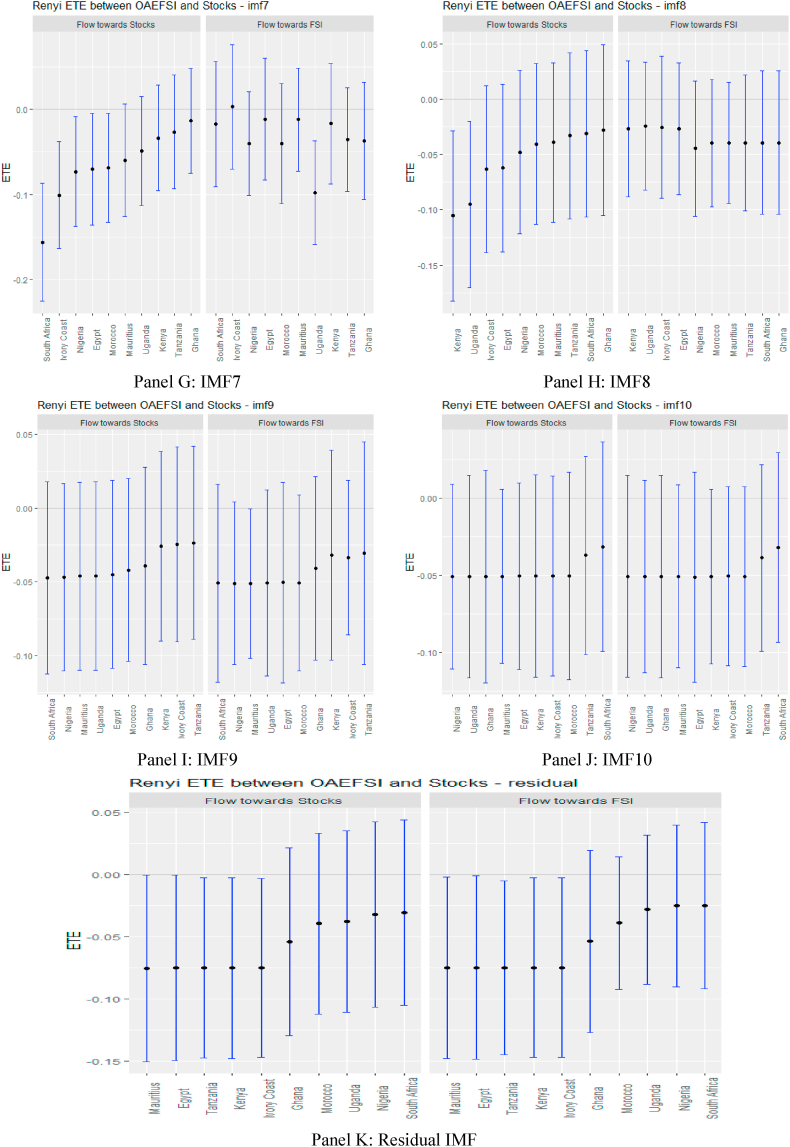
Information flow between other advanced economies' financial stress and African stock markets (Full sample). *Notes*: Panels A–J show the transfer entropies between the OAEFSI and African stocks across IMFs1-10, respectively while Panel K is for the residual IMF. The sample period falls between January 04, 2011 and March 10, 2022.

Turning to OAEFS in Fig. 3, we observe that the information flow to Egypt, Morocco, and the Ivory Coast is positive, suggesting that the equity markets in these economies are less risky in the short term. Ghana, Kenya, South Africa, and Uganda were found to be short-term negative recipients of ETE. This implies that these markets are highly risky for investment in the short term. The pattern of information flow across multiple scales remains the same, as we observed an increase in negative ETEs in eight African markets, except Ghana and Uganda. This implies that Africa's equity markets are soaked in stress from global market stress.

African markets are highly dependent on international economies for most of their economic activities and it is not surprising to see that the largest equity market in terms of market capitalisation in Africa (South Africa) appears to be integrated into the global market. It is also important to note that the flows of equity investment into South Africa come largely from the United States [71]. Indeed, South Africa is the most representative of African stock markets and has a strong link to advanced economies; therefore, the high stress on the GFS market negatively affects the performance of equity returns in South Africa. The increasing flow of information to South Africa from short to long term confirms the strong economic and financial relationship between South Africa and the global economy.

The persistence of negative ETEs due to international market stress in African stock markets establishes market inefficiency and can be delineated as financial contagion [72]. The effect of GFS information from different IMFs on African stock markets suggests that African financial markets remain weak.

### 5.2 Pre-COVID-19 pandemic period

**Fig. 4:**
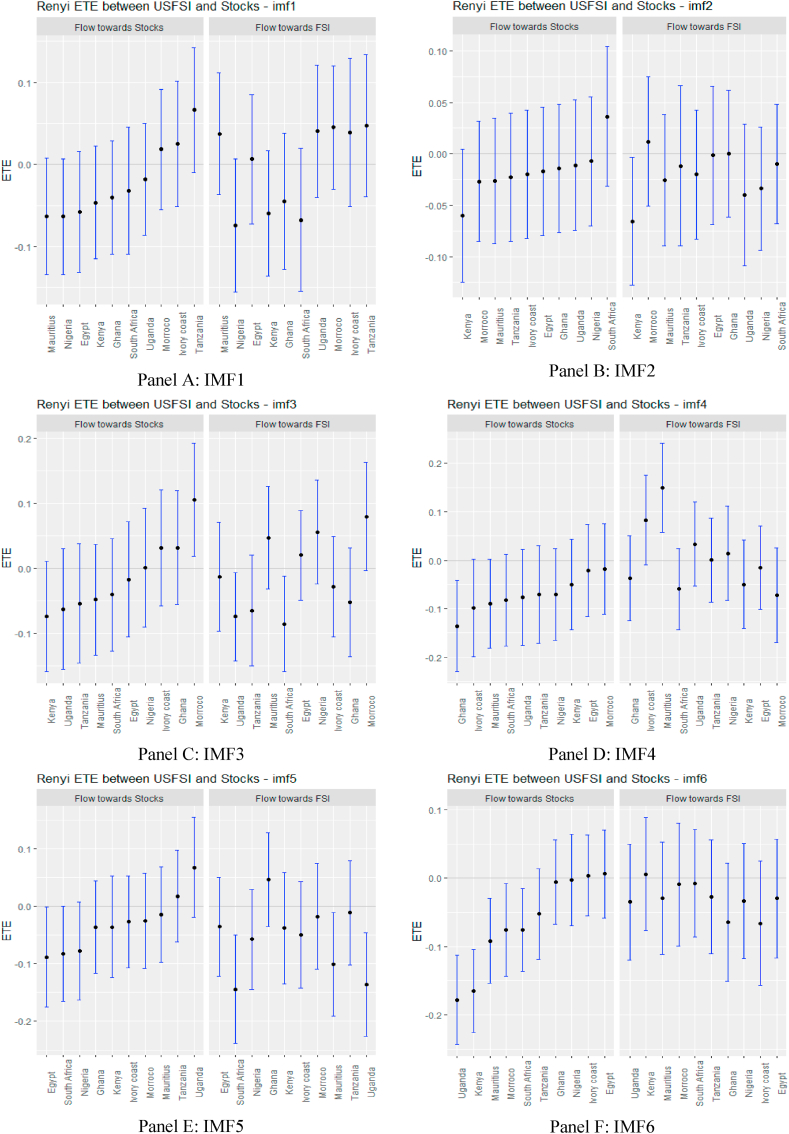

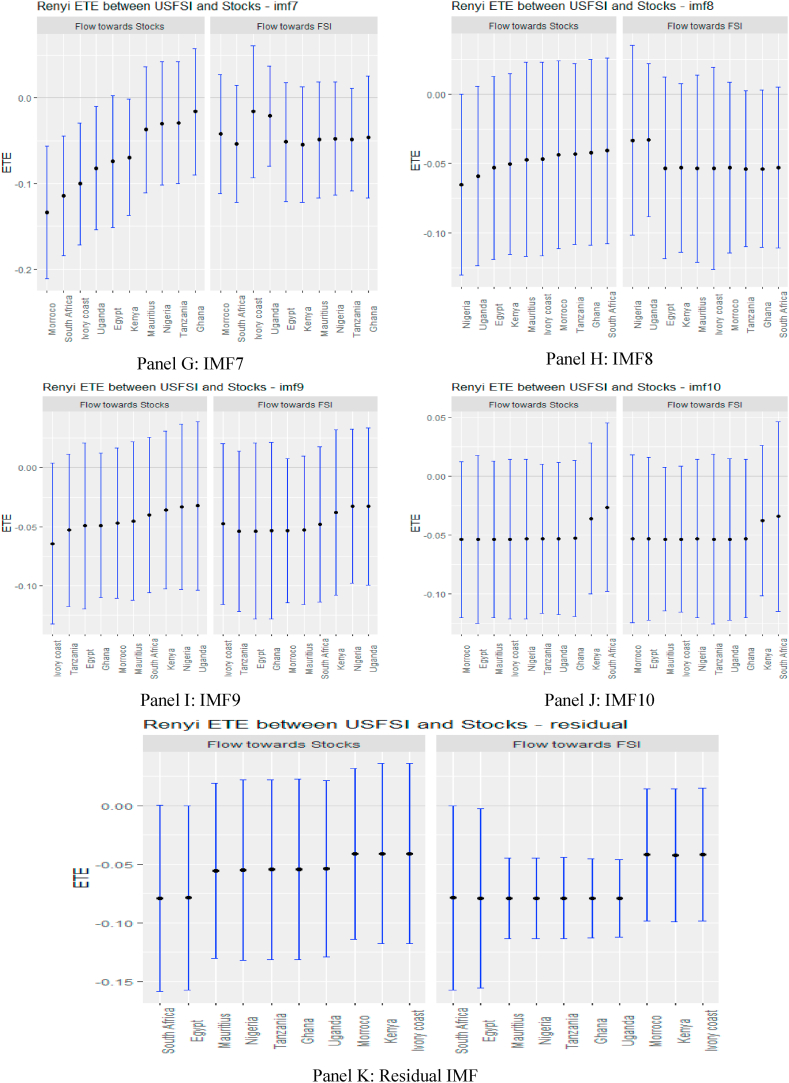
Information flow between the US financial stress and African stock markets (pre-COVID-19). *Notes*: Panels A–J show the transfer entropies between the USFSI and African stocks across IMFs1-10, respectively while Panel K is for the residual IMF. The sample period falls between January 04, 2011 and March 10, 2020.

A glance at the graphs in Fig. 4, supported numerically by the results in Table A2 (see Appendix), depicts that in the pre-COVID-19 pandemic period, USFSI ETEs were insignificant in the short term except for Ghana and Egypt which exhibited significant negative transfer entropies across IMFs 1–5. At the same time, stock returns were less risky for South Africa. Notwithstanding the insignificant information flow in the short term, the pattern of information flow within each market economy has deviated significantly, with Egypt responding to negative ETE in the short term when we screened the pandemic period from the entire sample. Therefore, the impact of the pandemic on financial markets could be envisaged [73].

**Fig. 5:**
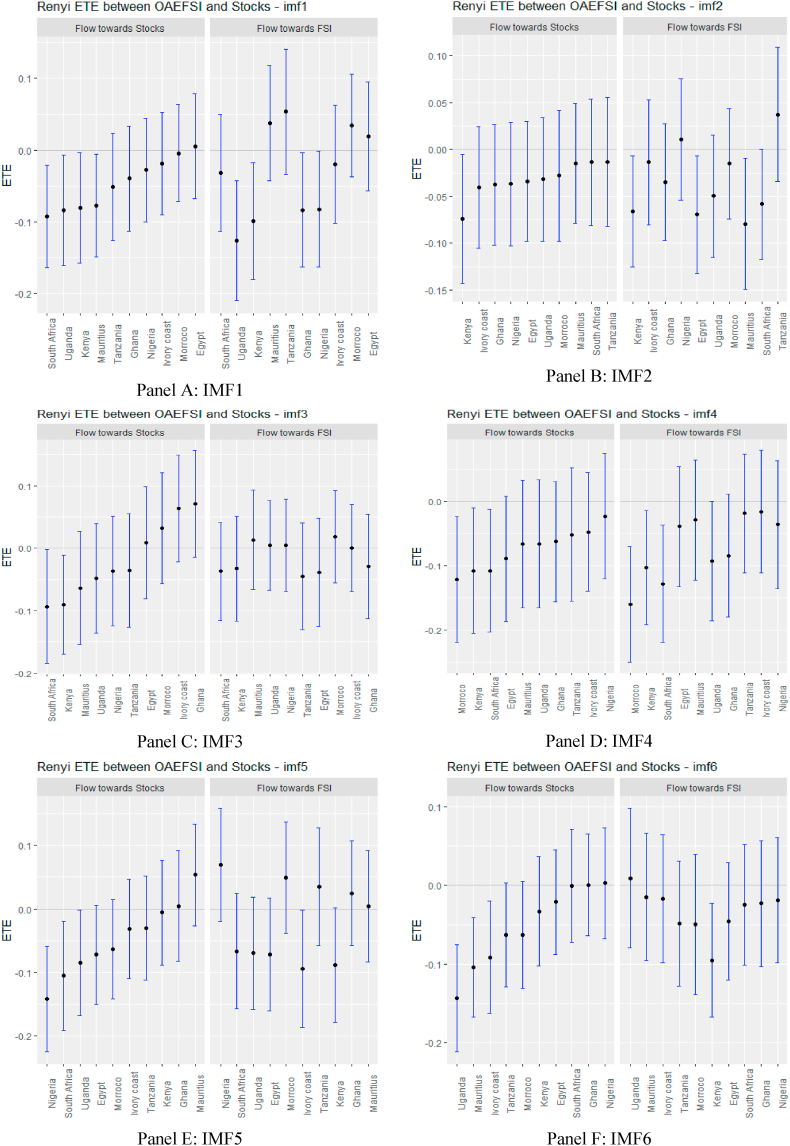

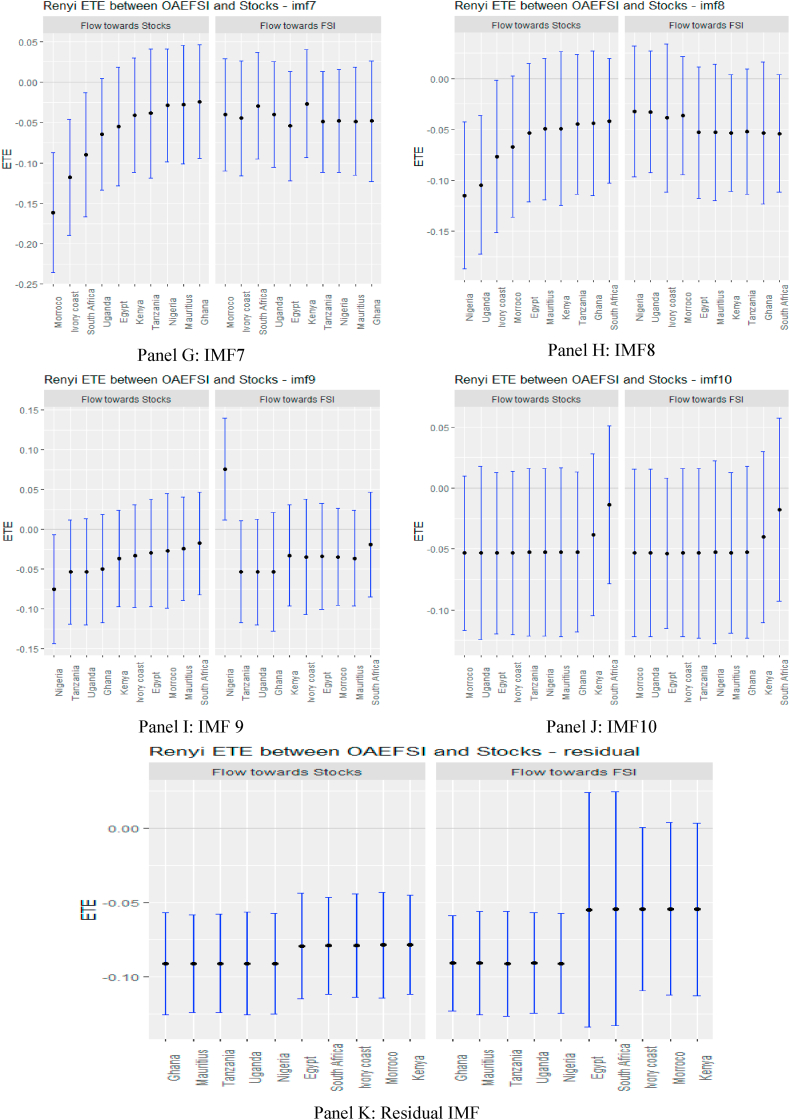
Information flow between other advanced economies' financial stress and African stock markets (pre-COVID-19). *Notes*: Panels A–J show the transfer entropies between the OAEFSI and African stocks across IMFs1-10, respectively while Panel K is for the residual IMF. The sample period falls between January 04, 2011 and March 10, 2020.

In the medium and long term, the pattern of information flow from USFSI did not change significantly, as Uganda, Kenya, Mauritius, Morocco, South Africa and Nigeria were significantly highly risky, but connoted diversification prospects for these markets. Focusing on OAEFSI in Fig. 5, we observe that the pattern of information flow deviates significantly in the long term. The count of significant information from OAEFSI increased in the long term, suggesting that all 10 African stock markets were significantly risky for investment. The time-scale-dependent diversification of equity investments amid international market stress partially supports the conclusion drawn by Maghyereh et al. [81]. Therefore, we note that financial liberalisation, financial globalisation, and market integration increase the instability of the equity market, and therefore financial markets are more prone as a result of the fundamental links between markets [74]. The increased significance of transfer entropy in the long term supports the assertion posited by Eom et al. [23], that financial markets’ degree of efficiency plays a pivotal role in the determination and direction of information flow and concluded that information flow is propagated by more efficient markets to their less efficient counterparts.

### 5.3 COVID-19 pandemic sample

**Fig. 6:**
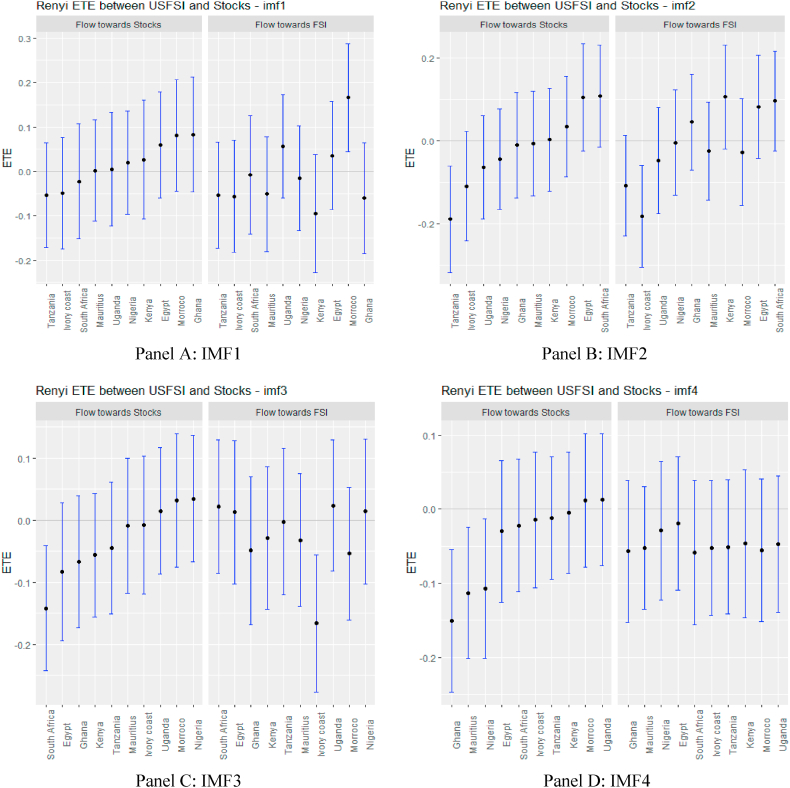

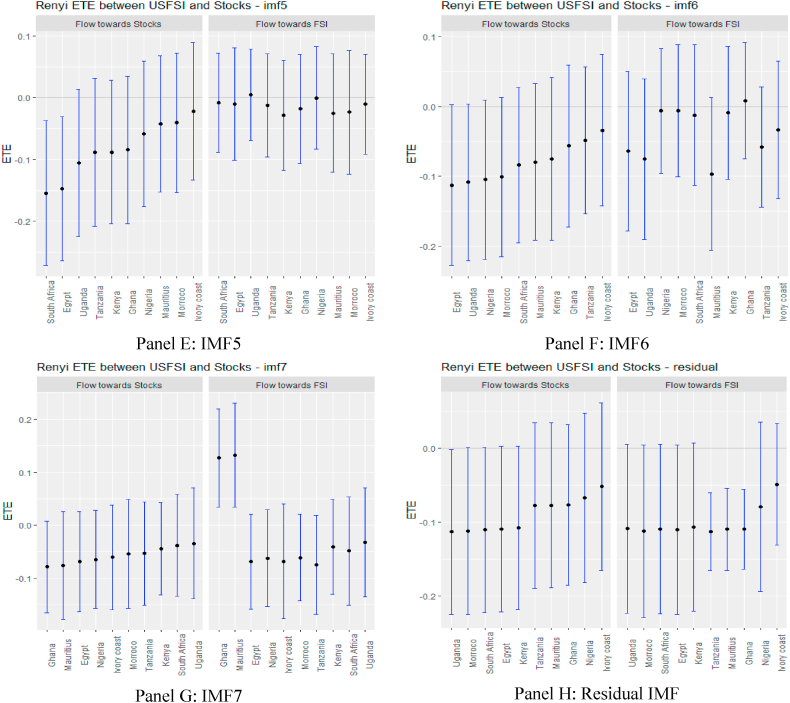
Information flow between the US financial stress and African stock markets (COVID-19 sample). *Notes*: Panels A–G show the transfer entropies between the USEFSI and African stocks across IMFs1-10, respectively while Panel H is for the residual IMF. The sample period falls between March 11, 2020 and March 10, 2022.

**Fig. 7:**
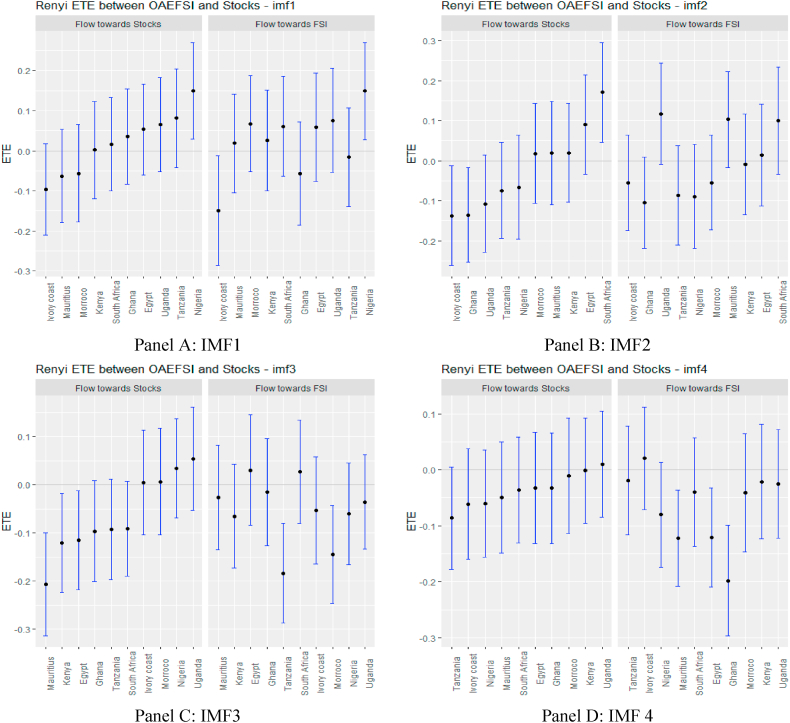

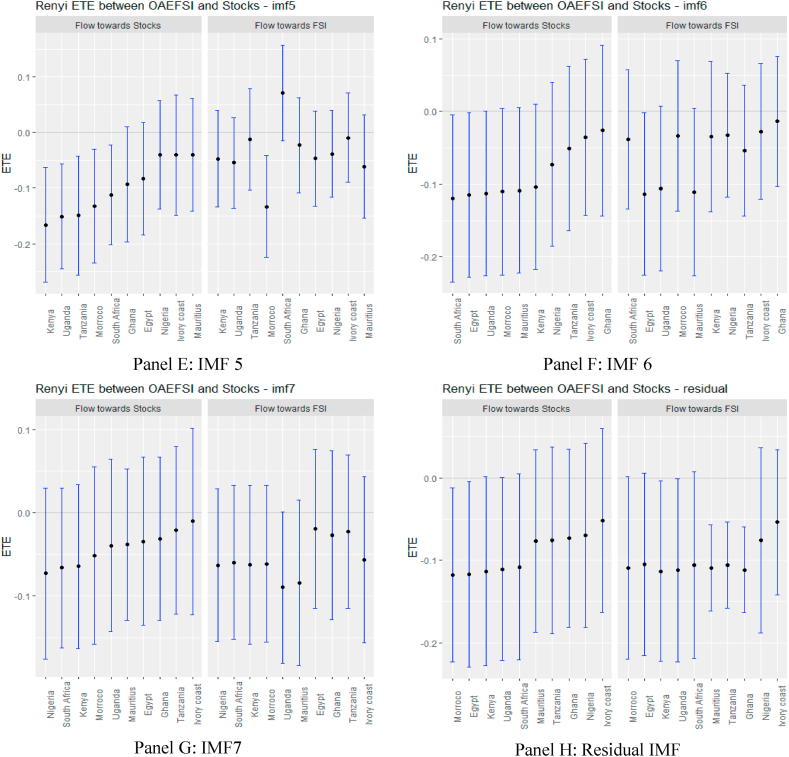
Information flow between other advanced economies' financial stress and African stock markets (COVID-19 sample). *Notes*: Panels A–G show the transfer entropies between OAEFSI and African stocks across IMFs1-10, respectively while Panel H is for the residual IMF. The sample period falls between March 11, 2020 and March 10, 2022.

The ETEs in Fig. 6, supported numerically by the results in Table A3 (see Appendix), show that during the sampled period of the COVID-19 pandemic, most of the short-term ETEs were insignificant except for Tanzania and South Africa, which are statistically significant. This is an indication that in the early days of the pandemic, several markets in Africa did not feel the impact severely due to the low market stress of the USFSI. This explains why Agyei et al. [75], explained that the effect of the pandemic on African economies had yet to manifest, but could have a long-term effect. Consistent with this, our medium-term ETEs reveal an interesting finding. As the pandemic reaches its peak when most markets are locked down, the stress of the financial markets intensified because there were no capital inflows or outflows. This exacerbated mutual information spillovers, presaging a significant high-risk status for the 10 African stock markets in the medium term and connoted zero diversification between multiple equity investments in Africa. The increase in ETEs over time horizons could be the consequence of financial contagion [76]. However, on the long-term residual scale, we notice that there is no discernible information flow in African markets as ETE disappears. This is due to the prevalent news regarding the pandemic, as investors may have calmed down to realise a more balanced portfolio in the long run, and hence the information flow from GFS may not have a significant impact in the long run. This pattern of information flow mirrors Fama's [77] EMH, which asserts that players in the financial market act rationally to demonstrate long-term market efficiency. Note that in the case of OAEFSI, as shown in Fig. 7, and Table A3, in the Appendix, it is not quite different from the USFSI information flow during the pandemic period.

## 6 Conclusions

This study quantifies the dependence between global financial market stress and African equity markets using daily data for the stock markets of 10 African countries, namely, Ghana Stock Exchange All Share Index (Ghana), Nigeria Stock Exchange All Share Index (Nigeria), Egypt EGX30 (Egypt), Casablanca All Share Index (Morocco), Nairobi All Share Index (Kenya), FT/JSE All Share Index (South Africa), Uganda Stock Exchange All Share Index (Uganda), Tanzania Stock Exchange All Share Index (Tanzania), Mauritius Stock Exchange All Share Index (Mauritius) and Cote D'Ivoire Stock Exchange All Share Index (Ivory coast) from January 04, 2011 to March 10, 2022. The study uses daily US financial stress indexes and other advanced economies' financial stress indexes to proxy global financial market stress. To overcome the problem of noisy data, we employ Ensemble Empirical Mode Decomposition (EEMD) to delineate the signal data into intramode functions (IMFs) that are representative of trading horizons (namely, short-, medium-, and long-term). We further employed transfer entropy to quantify the direction and strength of the information flow from the probability density function. We set the weighting parameter at (*q* = 0.3) to account for tailed events within the series.

Our findings underscore a substantial idiosyncrasy in the way equity markets in Africa are affected by the information flow from global market stress, and these yield a diversification advantage based on investment horizons or scales. The findings indicate that during the sampled era of COVID-19, African equity markets were found to be highly risky in the medium term when the transmission of shock emanates from USFSI or OAEFSI. The magnitude of information flow and its impact on equity performance in African markets during COVID-19 provide evidence of financial contagion [76]. Our findings highlight the established market dynamics during the pre-COVID-19 period in the long term, as we notice that there is a prospect of diversification for Ghana and Egypt in the short term and Tanzania, Cote D'Ivoire, and Egypt in the medium term.

The findings of the study reveal that an increase in market stress in the global economy holds significant information that investors, portfolio managers, and policymakers could rely on. For investors and portfolio managers, the dynamics of the GFS information flow signal the need to strategise investment decisions across investment horizons, particularly in pandemics like COVID-19. In particular, the applied methodology, i.e., the transfer entropy based on the EEMD, provides new insights into potential diversification advantages associated with African stocks in the frequency domain during stressed market conditions of the COVID-19 pandemic era. Diversification is informed by the combinations of low-risk and high-risk (positive and negative ETEs) which will enable investors to make decisions across investment horizons. Therefore, the findings are indispensable for investors who trade along timeliness designated as short-, medium-, and long-term investment horizons. Meanwhile, our findings suggest that maintaining multiple investments in African equities in the medium term during COVID-19 may not achieve diversification, as most African markets offer a similar response to stressed conditions. Investors and policymakers should take advantage of the results of the information flow from the GFS to African equity markets during economic uncertainty for effective portfolio management. Policymakers should be aware of economic events that affect marketability and adopt policies to mitigate the magnitude of the direction of information flow during global market stress, thus limiting the chances of financial contagion.

This study is limited to financial market stress from developed markets; therefore, for future research, financial market stress from emerging markets may be used to investigate the spillover effect of African frontier markets using frequency-dependent quantile regression during bullish, normal, and bearish trading states. Other quantile regression approaches [[78], [79], [80], [81]], could be considered. Importantly, given that Rényi effective entropy has been proposed relatively early, we recommend for future studies the application of new entropy features, such as dispersion entropy-based Lempel-Ziv complexity [82], particle swarm optimisation fractional slope entropy [83], and dispersion entropy-based Lempel-Ziv complexity [84] that may have a better distinguishing ability.

## Author contribution statement

Mohammed Armah: Conceived and designed the experiment; Performed and experiments; Analyzed and interpreted data; Contributed reagents, materials, analysis tools or data; Wrote the paper.

Ahmed Bossman: Performed and experiments; Analyzed and interpreted data; Contributed reagents, materials, analysis tools or data; Wrote the paper.

Godfred Amewu: Conceived and designed the experiments; Analyzed and interpreted data; Contributed reagents, materials, analysis tools or data; Wrote the paper.

## Funding statement

This research did not receive any specific grant from funding agencies in the public, commercial, or not-for-profit sectors.

## Data availability statement

Data will be made available on request.

## Declaration of interest’s statement

The authors declare no conflict of interest.

## Figures and Tables

**Table A1:** ETEs for the full sample.

Direction of flow	IMF1		IMF2		IMF3		IMF4		IMF5		IMF6		IMF7		IMF8		IMF9		IMF10		Residuals	
	ETE	SE	ETE	SE	ETE	SE	ETE	SE	ETE	SE	ETE	SE	ETE	SE	ETE	SE	ETE	SE	ETE	SE	ETE	SE
USFSI - > Egypt	−0.003	0.039	0.004	0.034	0.002	0.048	−0.040	0.062	−0.004	0.041	−0.024	0.037	−0.100	0.039	−0.035	0.039	−0.048	0.041	−0.015	0.036	−0.075	0.046
USFSI - > Ghana	0.018	0.040	−0.026	0.031	−0.036	0.049	−0.109	0.055	−0.091	0.043	0.016	0.041	−0.013	0.035	−0.029	0.043	−0.051	0.036	−0.050	0.038	−0.075	0.045
USFSI - > Ivory Coast	0.060	0.041	0.003	0.031	0.060	0.047	−0.007	0.058	−0.014	0.044	−0.094	0.043	−0.015	0.036	−0.034	0.040	−0.023	0.038	−0.038	0.036	−0.057	0.045
USFSI - > Kenya	−0.015	0.039	−0.034	0.032	−0.065	0.047	−0.049	0.056	−0.076	0.048	−0.117	0.039	−0.054	0.040	−0.055	0.039	−0.047	0.036	−0.051	0.040	−0.075	0.044
USFSI - > Mauritius	−0.070	0.042	−0.008	0.033	0.032	0.047	−0.065	0.058	−0.038	0.045	−0.030	0.039	−0.049	0.040	−0.039	0.038	−0.028	0.038	−0.049	0.039	−0.059	0.043
USFSI - > Morocco	0.055	0.038	0.022	0.032	0.003	0.050	−0.014	0.057	−0.001	0.044	−0.076	0.040	−0.124	0.035	−0.042	0.038	−0.041	0.040	−0.050	0.039	−0.039	0.044
USFSI - > Nigeria	−0.044	0.040	−0.005	0.034	0.014	0.049	−0.033	0.056	−0.018	0.048	−0.060	0.040	−0.066	0.038	−0.037	0.038	−0.044	0.042	−0.031	0.038	−0.032	0.044
USFSI - > South Africa	−0.045	0.042	−0.001	0.036	−0.042	0.053	−0.056	0.058	−0.165	0.046	−0.085	0.039	−0.172	0.038	−0.032	0.042	−0.047	0.034	−0.051	0.040	−0.030	0.044
USFSI - > Tanzania	0.017	0.039	0.034	0.037	−0.018	0.049	−0.067	0.058	0.054	0.043	0.017	0.040	−0.029	0.039	−0.029	0.039	−0.040	0.038	−0.051	0.039	−0.075	0.044
USFSI - > Uganda	−0.036	0.040	0.010	0.035	−0.042	0.049	−0.029	0.058	−0.075	0.044	−0.067	0.041	−0.150	0.035	−0.052	0.041	−0.045	0.036	−0.050	0.042	−0.037	0.046
OAEFSI - > Egypt	0.026	0.041	−0.035	0.033	0.002	0.048	−0.056	0.056	−0.096	0.048	−0.072	0.042	−0.070	0.040	−0.062	0.046	−0.045	0.039	−0.051	0.037	−0.075	0.045
OAEFSI - > Ghana	−0.049	0.039	−0.059	0.036	0.109	0.047	−0.073	0.058	−0.027	0.050	0.012	0.037	−0.014	0.038	−0.028	0.047	−0.039	0.041	−0.051	0.042	−0.054	0.046
OAEFSI - > Ivory Coast	0.008	0.042	−0.004	0.033	0.081	0.047	−0.033	0.059	−0.041	0.050	−0.025	0.039	−0.101	0.038	−0.063	0.046	−0.025	0.040	−0.050	0.040	−0.075	0.044
OAEFSI - > Kenya	−0.069	0.043	−0.021	0.032	−0.062	0.048	−0.099	0.055	−0.098	0.048	−0.088	0.039	−0.034	0.038	−0.105	0.047	−0.026	0.039	−0.050	0.040	−0.075	0.044
OAEFSI - > Mauritius	−0.074	0.041	0.001	0.033	−0.033	0.048	0.063	0.059	−0.032	0.047	−0.098	0.039	−0.060	0.040	−0.039	0.044	−0.046	0.039	−0.051	0.034	−0.075	0.046
OAEFSI - > Morocco	0.080	0.039	−0.011	0.033	0.049	0.048	−0.064	0.056	−0.091	0.048	−0.032	0.042	−0.069	0.039	−0.041	0.044	−0.042	0.038	−0.050	0.041	−0.039	0.044
OAEFSI - > Nigeria	−0.041	0.041	−0.015	0.034	0.017	0.048	−0.047	0.055	−0.044	0.047	−0.016	0.042	−0.073	0.039	−0.048	0.045	−0.047	0.039	−0.051	0.036	−0.032	0.045
OAEFSI - > South Africa	−0.069	0.039	0.009	0.032	−0.063	0.048	−0.046	0.059	−0.039	0.052	−0.023	0.040	−0.156	0.042	−0.031	0.046	−0.047	0.040	−0.031	0.041	−0.031	0.045
OAEFSI - > Tanzania	0.021	0.043	0.052	0.034	0.050	0.048	−0.065	0.058	−0.019	0.048	−0.014	0.040	−0.027	0.041	−0.033	0.046	−0.024	0.040	−0.037	0.039	−0.075	0.044
OAEFSI - > Uganda	−0.063	0.040	−0.015	0.034	−0.027	0.046	−0.066	0.057	−0.182	0.049	−0.105	0.041	−0.049	0.039	−0.095	0.046	−0.046	0.039	−0.051	0.040	−0.038	0.044

**Notes:** ETE, SE, IMF, USFSI, and OAEFSI denote estimate transfer entropy, standard error, intrinsic mode function, the US financial stress index, and other advanced economies' financial stress indexes.

**Table A2:** ETEs for the pre-COVID-19 sample.

Direction of flow	IMF1		IMF2		IMF3		IMF4		IMF5		IMF6		IMF7		IMF8		IMF9		IMF10		Residuals	
	ETE	SE	ETE	SE	ETE	SE	ETE	SE	ETE	SE	ETE	SE	ETE	SE	ETE	SE	ETE	SE	ETE	SE	ETE	SE
USFSI - > Egypt	−0.058	0.045	−0.017	0.038	−0.017	0.054	−0.021	0.058	−0.088	0.053	0.006	0.039	−0.074	0.047	−0.053	0.040	−0.049	0.043	−0.054	0.043	−0.079	0.048
USFSI - > Ghana	−0.040	0.042	−0.014	0.038	0.032	0.053	−0.136	0.058	−0.037	0.049	−0.006	0.037	−0.016	0.045	−0.042	0.041	−0.049	0.037	−0.053	0.040	−0.054	0.047
USFSI - > Ivory Coast	0.025	0.046	−0.020	0.038	0.031	0.054	−0.099	0.061	−0.027	0.049	0.004	0.036	−0.100	0.043	−0.047	0.043	−0.064	0.041	−0.053	0.041	−0.041	0.047
USFSI - > Kenya	−0.046	0.042	−0.060	0.039	−0.074	0.051	−0.050	0.056	−0.036	0.054	−0.165	0.037	−0.069	0.041	−0.050	0.040	−0.036	0.041	−0.036	0.039	−0.041	0.047
USFSI - > Mauritius	−0.064	0.043	−0.026	0.037	−0.048	0.052	−0.090	0.056	−0.015	0.050	−0.092	0.038	−0.037	0.045	−0.047	0.042	−0.045	0.041	−0.054	0.040	−0.055	0.045
USFSI - > Morocco	0.019	0.044	−0.027	0.036	0.105	0.053	−0.018	0.057	−0.025	0.050	−0.075	0.041	−0.134	0.047	−0.044	0.041	−0.047	0.039	−0.054	0.040	−0.041	0.044
USFSI - > Nigeria	−0.063	0.043	−0.007	0.038	0.001	0.056	−0.071	0.058	−0.078	0.052	−0.003	0.040	−0.030	0.044	−0.065	0.040	−0.033	0.043	−0.053	0.041	−0.055	0.047
USFSI - > South Africa	−0.032	0.047	0.036	0.041	−0.041	0.053	−0.082	0.058	−0.082	0.050	−0.075	0.037	−0.114	0.043	−0.041	0.041	−0.040	0.040	−0.026	0.043	−0.079	0.048
USFSI - > Tanzania	0.066	0.046	−0.023	0.038	−0.054	0.056	−0.071	0.061	0.017	0.049	−0.052	0.040	−0.029	0.043	−0.043	0.040	−0.053	0.039	−0.053	0.038	−0.054	0.047
USFSI - > Uganda	−0.018	0.041	−0.011	0.039	−0.063	0.056	−0.076	0.060	0.067	0.053	−0.178	0.040	−0.082	0.044	−0.059	0.039	−0.032	0.043	−0.053	0.039	−0.054	0.046
OAFSI - > Egypt	0.006	0.045	−0.034	0.039	0.009	0.055	−0.089	0.059	−0.072	0.047	−0.021	0.041	−0.055	0.045	−0.053	0.041	−0.029	0.041	−0.053	0.040	−0.079	0.022
OAFSI - > Ghana	−0.040	0.045	−0.038	0.039	0.071	0.052	−0.062	0.057	0.005	0.053	0.001	0.039	−0.024	0.043	−0.044	0.043	−0.049	0.041	−0.053	0.040	−0.091	0.021
OAFSI - > Ivory Coast	−0.018	0.043	−0.040	0.039	0.064	0.052	−0.048	0.056	−0.031	0.048	−0.091	0.043	−0.118	0.044	−0.077	0.045	−0.033	0.039	−0.053	0.041	−0.079	0.021
OAFSI - > Kenya	−0.081	0.047	−0.074	0.042	−0.090	0.048	−0.108	0.059	−0.005	0.050	−0.033	0.042	−0.041	0.043	−0.049	0.046	−0.036	0.037	−0.038	0.040	−0.078	0.020
OAFSI - > Mauritius	−0.077	0.043	−0.015	0.039	−0.064	0.055	−0.066	0.060	0.054	0.049	−0.104	0.039	−0.028	0.045	−0.050	0.042	−0.024	0.039	−0.053	0.042	−0.091	0.020
OAFSI - > Morocco	−0.004	0.041	−0.028	0.042	0.032	0.054	−0.121	0.060	−0.064	0.048	−0.063	0.042	−0.161	0.045	−0.067	0.042	−0.027	0.044	−0.054	0.038	−0.079	0.022
OAEFSI - > Nigeria	−0.028	0.044	−0.037	0.040	−0.037	0.053	−0.023	0.059	−0.142	0.051	0.003	0.043	−0.028	0.042	−0.115	0.044	−0.075	0.042	−0.053	0.042	−0.091	0.021
OAFSI - > South Africa	−0.092	0.044	−0.013	0.041	−0.094	0.056	−0.108	0.058	−0.105	0.052	0.000	0.044	−0.090	0.047	−0.042	0.037	−0.017	0.039	−0.014	0.039	−0.079	0.020
OAFSI - > Tanzania	−0.051	0.045	−0.013	0.042	−0.036	0.055	−0.051	0.063	−0.030	0.050	−0.063	0.040	−0.038	0.049	−0.045	0.042	−0.053	0.040	−0.053	0.042	−0.091	0.020
OAFSI - > Uganda	−0.083	0.047	−0.032	0.040	−0.048	0.053	−0.066	0.061	−0.085	0.051	−0.143	0.042	−0.064	0.042	−0.104	0.041	−0.053	0.040	−0.053	0.043	−0.091	0.021

**Notes:** ETE, SE, IMF, USFSI, and OAEFSI denote estimate transfer entropy, standard error, intrinsic mode function, the US financial stress index, and other advanced economies' financial stress indexes.

**Table A3:** ETEs for the COVID-19 sample.

Direction of flow	IMF1		IMF2		IMF3		IMF4		IMF5		IMF6		IMF7		Residuals	
	ETE	SE	ETE	SE	ETE	SE	ETE	SE	ETE	SE	ETE	SE	ETE	SE	ETE	SE
USFSI - > Egypt	0.059	0.073	0.105	0.079	−0.083	0.067	−0.030	0.058	−0.147	0.071	−0.112	0.070	−0.069	0.057	−0.109	0.068
USFSI - > Ghana	0.083	0.079	−0.010	0.077	−0.067	0.065	−0.150	0.058	−0.085	0.072	−0.056	0.071	−0.079	0.053	−0.077	0.066
USFSI - > Ivory Coast	−0.049	0.076	−0.110	0.080	−0.008	0.068	−0.014	0.056	−0.022	0.068	−0.034	0.066	−0.061	0.060	−0.052	0.069
USFSI - > Kenya	0.026	0.082	0.002	0.075	−0.056	0.061	−0.005	0.049	−0.088	0.071	−0.075	0.071	−0.045	0.053	−0.108	0.067
USFSI - > Mauritius	0.002	0.069	−0.007	0.077	−0.009	0.066	−0.113	0.054	−0.043	0.067	−0.080	0.068	−0.076	0.062	−0.077	0.068
USFSI - > Morocco	0.081	0.076	0.034	0.074	0.032	0.065	0.012	0.055	−0.041	0.069	−0.101	0.069	−0.055	0.063	−0.112	0.068
USFSI - > Nigeria	0.020	0.071	−0.045	0.074	0.035	0.062	−0.107	0.057	−0.058	0.072	−0.105	0.069	−0.065	0.056	−0.067	0.070
USFSI - > South Africa	−0.023	0.079	0.108	0.074	−0.142	0.061	−0.022	0.054	−0.154	0.071	−0.084	0.068	−0.038	0.058	−0.111	0.068
USFSI - > Tanzania	−0.054	0.072	−0.189	0.078	−0.045	0.065	−0.012	0.050	−0.088	0.073	−0.049	0.064	−0.054	0.060	−0.078	0.068
USFSI - > Uganda	0.005	0.078	−0.064	0.076	0.015	0.062	0.013	0.054	−0.105	0.072	−0.108	0.068	−0.035	0.064	−0.113	0.068
OAEFSI - > Egypt	0.053	0.069	0.090	0.075	−0.115	0.062	−0.033	0.060	−0.083	0.062	−0.115	0.069	−0.034	0.061	−0.117	0.068
OAEFSI - > Ghana	0.036	0.072	−0.135	0.072	−0.096	0.064	−0.033	0.060	−0.093	0.063	−0.026	0.071	−0.031	0.060	−0.073	0.066
OAEFSI - > Ivory Coast	−0.097	0.069	−0.137	0.076	0.005	0.066	−0.061	0.060	−0.040	0.066	−0.035	0.065	−0.010	0.068	−0.052	0.068
OAEFSI - > Kenya	0.002	0.074	0.020	0.075	−0.121	0.063	−0.001	0.057	−0.166	0.062	−0.104	0.069	−0.064	0.060	−0.113	0.070
OAEFSI - > Mauritius	−0.063	0.071	0.019	0.078	−0.206	0.065	−0.050	0.060	−0.040	0.061	−0.109	0.069	−0.038	0.055	−0.077	0.067
OAEFSI - > Morocco	−0.056	0.073	0.018	0.076	0.007	0.067	−0.010	0.063	−0.133	0.062	−0.110	0.070	−0.051	0.065	−0.118	0.064
OAEFSI - > Nigeria	0.150	0.073	−0.066	0.079	0.035	0.062	−0.060	0.058	−0.040	0.059	−0.073	0.068	−0.073	0.062	−0.070	0.068
OAEFSI - > South Africa	0.016	0.071	0.171	0.076	−0.091	0.060	−0.036	0.057	−0.112	0.054	−0.119	0.070	−0.066	0.058	−0.108	0.069
OAEFSI - > Tanzania	0.081	0.075	−0.075	0.073	−0.092	0.064	−0.086	0.056	−0.149	0.065	−0.051	0.069	−0.021	0.061	−0.076	0.069
OAEFSI - > Uganda	0.065	0.071	−0.107	0.074	0.055	0.065	0.010	0.057	−0.151	0.057	−0.113	0.068	−0.039	0.063	−0.111	0.068

**Notes:** ETE, SE, IMF, USFSI, and OAEFSI denote estimate transfer entropy, standard error, intrinsic mode function, the US financial stress index, and other advanced economies' financial stress indexes.
